# Genetic Variation in Interleukin 28B and Response to Antiviral Therapy in Patients with Dual Chronic Infection with Hepatitis B and C Viruses

**DOI:** 10.1371/journal.pone.0077911

**Published:** 2013-10-17

**Authors:** Xiaoyan Guo, Guilin Yang, Jin Yuan, Peng Ruan, Mingxia Zhang, Xincun Chen, Boping Zhou

**Affiliations:** 1 Department of Infectious Diseases, The Third Affiliated Hospital of Sun Yat-Sen University, Guangzhou, China; 2 Key Laboratory of Tropical Disease Control (Sun Yat-sen University), Ministry of Education, Guangzhou, China; 3 Institute of Hepatology, Shenzhen Third people’s Hospital, Shenzhen, China; Duke University, United States of America

## Abstract

Concurrent infection with hepatitis C virus (HCV) and hepatitis B virus (HBV) was not uncommon in China. To date, information on predictors of response to treatment of dually-infected HCV/HBV is limited. The aim of this study was to evaluated whether determination of the interleukin 28B (IL-28B) polymorphism statuses sufficient to predict treatment response of interferon (IFN)-based therapy in patients chronically infected with both hepatitis B and C viruses. We investigated the role of IL28B variations (rs8099917 and rs12979860) in response to IFN-based treatment and evaluated its association with the risk of the null virological response (NVR) in HCV /HBV dually-infected patients. We found that the overall distributions of the genotypes among the sustained virological response (SVR), NVR groups were significantly different (P<0.001): patients with the rs8099917 TG genotype had an increased risk of NVR (odds ratio [OR] =2.37 95% confidence interval [CI] =1.16–4.83, P =0.017), and those with the GG genotype had a further increased risk of NVR (OR=4.23, 95% CI =1.17-15.3, P=0.027). The rs12979860 allele was also highly associated with treatment failure (CT/TT vs. CC; OR =2.04, 95%CI =1.05-3.97, P =0.037). Moreover, we found that IL28B rs8099917 G variants (TG+GG) interact with HCV genotype 1(G1) to result in higher risk of NVR (P=0.009), and that they are also associated with HBV DNA reactivation (TG+GG vs. TT, P=0.005). Furthermore, multivariate regression analysis show that the rs8099917 G allele was the most important factor significantly associated with a NVR in HCV G1 patients. This study suggest that IL28B genotyping may be a valid pretreatment predictor of which patients are likely to respond to treatment in this group of difficult-to-treat HCV/HBV dually-infected patients.

## Introduction

Hepatitis B (HBV) and C virus (HCV) infections are the leading causes of chronic liver disease worldwide[1]. Both viruses share the same modes of transmission, dual infection with HBV/HCV is not uncommon in Asian patients [2-5]. Several cross-sectional studies found that patients dually infected with HBV/HCV has been associated with a more severe clinical course, including a significantly higher risk of developing cirrhosis or hepatocellular carcinoma (HCC), as compared with mono-infection alone[2,6-9]. Although there is no standard of care for HBV/HCV dually-infected patients, several studies have confirmed that combination therapy with peginterferon alfa and ribavirin(PEG-IFN-α/RBV) is equally effective in patients with HCV mono-infection[10-12]. Previous studies have shown that sustained HCV clearance by PEG-IFN-α/RBV therapy may significantly reduce HCC in HBV/HCV dually-infected patients [13,14]. Due to their distinct clinical course and heterogeneity of the dually-infected patient population, prediction of the probable effectiveness of IFN therapy therefore plays an essential role in the clinical setting. 

Recently, some independent genome-wide association studies have identified that several genetic polymorphisms of the IL28B gene (also known as IFN-λ3), such as rs8099917 and rs12979860, effectively predicts responses in patients with chronic hepatitis C infections treated with PEG-IFN-α/RBV[15-17]. The findings also have been confirmed in other populations, including chronic hepatitis B [18,19]and chronic hepatitis C patients who are also co-infected with human immunodeficiency virus (HIV)[20]. Furthermore, the type of IFN coded for by IL28B, IFN-λ has been shown previously to be active against HBV DNA viruses[21,22]. It is likely that this relationship is not specific to patients with HCV infection. Whether IL28B polymorphisms are also related to response to PEG-IFN treatment in HBV/HCV dually- infected patients is currently unknown.

In this study, we attempted to investigate the prevalence and the effect on response to PEG-IFN-α/RBV treatment of two IL28B gene polymorphisms (rs8099917 and rs12979860) in patients with dual HBV/HCV infection.

## Patients and Methods

### Patients

The study protocol was approved by the institutional review boards of Sun Yat-Sen University. Written informed consent was obtained from each participant after a full explanation of the study.

We retrospectively recruited 146 Chinese patients with dual HBV/HCV infection who underwent the PEG-IFN-α/RBV therapy between 2009 and 2011 in the outpatient clinic of the Third Affiliated Hospital of Sun Yat-Sen University or the Institute of Hepatology, Shenzhen Third people’s Hospital. Dual infection was defined by seropositivity both for antibodies to HCV (anti-HCV) and for HB surface antigen (HBsAg) for more than 6 months together with a serum HCV-RNA level of 200 IU/mL or higher (1000 copies/mL)[23]. Patients were excluded if they had hepatitis A, D or E, or HIV infection. Further exclusion criteria included autoimmune disease, psychiatric disease, uncontrolled diabetes mellitus, symptomatic cardiac or cardiovascular disease, decompensated liver disease and hepatocellular carcinoma.

Treatment guidelines in existence for patients with chronic hepatitis C and chronic hepatitis B mono-infection can be applied to dually-infected patients[23,24]. All eligible subjects were treated with PEG-IFN-α-2a at a fixed dose of 180μg/week and ribavirin 800-1,200mg/day (i.e., 800mg for patients <65 kg; 1,000 mg for patients weighing 65 to 85 kg; 1,200 mg for patients weighing 85 to 105 kg). Patients with HCV genotype 1 and 6 were treated for 48weeks and patients with HCV genotype 2 or 3 were treated for 24 weeks. After completing the therapy, the patients were followed- up for 24 weeks and the therapeutic effectiveness was evaluated. Patients included in the SVR group had normal alanine aminotransferase (ALT) levels and no evidence of viremia 24 weeks after completion of IFN therapy. Those included in the NVR group had HCV RNA levels detectable during the completed period of the treatment. Reactivation of HBV DNA was defined as 200 international units (IU)/ml detected after the week 24 posttreatment. Ultrasound guided percutaneous liver biopsies were performed for 21 patients. All biopsy specimens were used for the histologic diagnosis. Disease staging was defined according to Desmet[17,25] with ranking from F0 (absence of fibrosis) to F4 (cirrhosis stage). 

### Serological and virological assays

HBsAg, HBeAg, antibody to HBeAg (anti-HBe) and anti-HDV were assayed using commercially available enzyme immunoassay kits (Abbott Laboratories, North Chicago, IL, USA). Serum HBV DNA was quantified with a sensitive polymerase chain reaction (PCR) assay (COBAS Amplicor HBV Monitor, Roche Diagnostics GmbH, Mannheim, Germany; with a lower quantitation limit of 1000 copies/ml (200 IU/ml). Dilution was performed if HBV DNA levels were more than 10^6^ copies/ml. HBV genotyping was performed in patients by restriction fragment length polymorphism. The serum HCV-RNA level was determined by real-time PCR assay, with a lower limit of detection of 50 IU/mL (COBAS TaqMan HCV Testversion 2.0; Roche Diagnostics), and HCV genotype using the InnoLipa genotyping kit (Innogenetics, Zwijndrecht, Belgium).

### Genomic DNA Isolation and IL-28B genotyping

Genomic DNA was extracted from 200 μL of the cell suspension with Omega kits (Omega-Tek, Mansﬁeld, OH, USA) according to the manufacturer’s instructions. The PCR bands of interest were excised from the agarose gel, and the DNA fragments were purified with gel extraction kits (Omega-Tek). Purified genomic DNA (10-20ng) was used for genotyping. TaqMan SNP Genotyping Assays(Applied Biosystems, Foster City, CA, USA) were used for the detection two the SNP, rs8099917 and rs12979860, near the IL28B gene on chromosome 19, using the following sequencing primers from Shanghai Invitrogen Biotechnology Co.,Ltd.(Beijing, China): for rs8099917, sense,5'-CCCACTTCTGGAACAAATCGTCCC-3'anti-sense, 5'-TCAAC

CCCACCTCAAATTATCCTA-3'; and for rs12979860, sense, 5'-GCCTGTCGTGTACTGAACCA-3';and anti-sense, 5'GCGCGGAGTGCAATTCAAC-3.Allele discrimination was achieved by detecting fluorescence using System SDS software (Applied Biosystems, Foster City, CA, USA).

### Statistical analysis

Frequencies were compared between groups using either a chi-square test with the Yates correction or the Fisher exact test. Group means, presented as mean values and standard deviations, were compared using analysis of variance and the Student’s t-test or Mann–Whitney U test. To assess the relative contribution of predictors of SVR and NVR, the odds ratios (ORs) and 95% confidence intervals (CIs) were calculated. All statistical analysis was performed using SPSS version16.0 software (SPSS Inc, Chicago, IL, USA) and SAS 9.2 (SAS Institute Inc, Cary, NC). All statistical analyses were based on two-sided hypothesis tests with a significance level at p<0.05.

## Results

### Baseline characteristics

The clinical characteristics and the treatment responses of the146 HBV/HCV dually -infected patients in the study are presented in Table 1. Overall, there was no significant difference in our study cohort between the SVR and NVR groups with respect to age, gender, body mass index(BMI), liver enzyme levels, platelet count ,HCV RNA viral load , HBV genotype, and fibrosis stage (all *P*>0.05). However, the HCV genotype frequencies were significantly different between the NVR and SVR groups (*P*=0.01). HBV DNA viral load was higher in the NVR groups as compared with the SVR groups (P=0.001). Remarkable differences were also detected in differences HBeAg status, HBsAg-positive patients were more predominant in the NVR group (P=0.042). The genotype distribution of HBV was B in 59 (40%), C in 73 (50%) and non-classified in 14 (10%) of the patients due to undetectable viral genomes or too-weak signals of PCR products for further genotyping. 

**Table 1:** Clinical Characteristics of patients with chronic HBV and HCV dual-infection and treated with peg-IFN-a/RBV therapy.

Variable	SVR (n=84)	NVR(n=62)	*P* value
Age	44.5±11.4	43.5±12.4	0.612
Gender			0.788
Male	51(60.7%)	39(62.9%)	
Female	33(39.3%)	23(37.1%)	
Body mass index, kg/m^2^	22.25±2.70	22.20±3.00	0.682
AST(IU/L)	64(10-356)	86(13-659)	0.205
ALT(IU/L)	70(17-197)	72(16-540)	0.597
Platelet count (×10^4^/mm^3^)	17.69±5.56	16.41±5.43	0.166
Fibrosis stage^*^			0.325
F0-F1	2(15.4%)	2(25.0%)	
F2	9(69.2%)	3(37.5%)	
F3	1(7.7%)	1(12.5%)	
F4	1(7.7%)	2(25.0%)	
Cirrhotic^$^			0.367
Yes	9(12.7%)	10(18.5%)	
No	62(87.3%)	44(81.5%)	
HBV DNA			0.001
Detectable(≥200IU/ml)	29(34.5%)	38(61.3%)	
Undetectable(<200IU/ml)	55(65.5%)	24(38.7%)	
HBV genotype			0.219
B	34(40.5%)	25(40.4%)	
C	39(46.4%)	34(54.8%)	
Non-classiﬁed	11(13.1%)	3(4.8%)	
HBeAg State			0.042
Negative	68(81.0%)	41(66.1%)	
Positive	16(19.0%)	21(33.9%)	
HCV genotype			0.010
G1	39(46.4%)	42(67.7%)	
Non-G1	45(53.6%)	20(32.3%)	
HCV RNA (copies/ml)( Log_10_)	6.16±1.16	6.06±1.03	0.580

ALT, AST, Median, Mann-Whitney U test; Gender, Fibrosis stage, HBV DNA, HBV Genotype, HBeAb State, and HCV genotype, Chi-square test.

* Only 21 patients had Fibrosis stage by ultrasound guided percutaneous liver biopsies, and the chi-square test performed between fibrosis stages F0-2 and F3-4.

$: Clinical diagnosis of cirrhosis was based on repeated ultrasound clinical criteria or signs of portal hypertension [33].

### The prevalence of IL28B genotypes with response to PEG-IFN-α/RBV treatment

The genotype and allele distributions of the IL28B rs8099917 and rs12979860 polymorphisms are summarized in Table 2. The overall distributions of the genotypes between the NVR and SVR groups were significantly different (P<0.05). For rs8099917, the TG+GG variant of rs8099917 SNP was associated with NVR (58.1% in the NVR group and 34.5% in the SVR group). Logistic regression analysis showed that compared with patients with the TT genotype, those with the TG genotype had an increased risk of NVR (odds ratio [OR] =2.37, 95%confidence interval [CI] =1.16–4.83, P=0.017), and the GG genotype was associated with a further increased risk of NVR (OR=4.23, 95%CI=1.17–15.33, P=0.027). Complicated TG with GG genotype together, G variant genotypes (TG+GG) also had an increased risk of NVR, and there was a significant trend for a dose-effect of the G allele on the risk of NVR. Moreover, compared with T allele carriers, G allele carriers had a significantly higher risk of NVR (OR= 2.25, 95%CI =1.33–3.82, P=0.002). For rs12979860, the CT+TT genotype was also associated with NVR (58.1% in NVR, 40.4% in SVR). Logistic regression analysis showed that, compared with patients with the CC genotype, the risk of NVR increased in those with the TT genotype (OR=5.77 95% CI=1.09–30.62, P=0.040), but the risk of NVR increased in those patients with the CT genotype was not observed (OR =1.80 95% CI=0.91-3.59, P=0.092). Complicated CT with TT genotype together, T variant genotypes (CT+TT) also had an increased risk of NVR, and there was a significant trend for a dose-effect of the T allele on the risk of NVR (OR= 2.04, 95%CI =1.05-3.97, P=0.037). Similarly, compared with C allele carriers, T allele carriers had a significantly higher risk of NVR (OR=1.88, 95% CI =1.11-3.17, P=0.018). In short, rs8099917 and rs12979860 were strongly linked with response to PEG-IFN-α/RBV treatment in HBV/ HCV dually-infected patients.

**Table 2:** The prevalence of IL28B genotypes with response to PEG-IFN-α/RBV treatment.

Genotype	SVR(n=84)	NVR(n=62)	OR (95% CI) NVR vs. SVR	*P*-value
rs8099917				
TT	55(65.5%)	26(41.9%)	1.00(reference)	
TG	25(29.8%)	28(45.2%)	2.37(1.16-4.83)	0.017
GG	4(4.7%)	8(12.9%)	4.23(1.17-15.33)	0.027
TG+GG	29(34.5%)	36(58.1%)	2.63(1.34-5.16)	0.005
T allele	135(80.4%)	80(64.5%)	1.00(reference)	
G allele	33(19.6%)	44(35.5%)	2.25(1.33-3.82)	0.002
rs12979860				
CC	50(59.6%)	26(41.9%)	1.00(reference)	
CT	32(38.1%)	30(48.4%)	1.80(0.91-3.59)	0.092
TT	2(2.3%)	6(9.7%)	5.77(1.09-30.62)	0.040
CT+TT	29(40.4%)	36(58.1%)	2.04(1.05-3.97)	0.037
C allele	132(78.6%)	82(66.1%)	1.00(reference)	
T allele	36(21.4%)	42(33.9%)	1.88(1.11-3.17)	0.018

### Treatment response according to IL28B genotype in patients with different HCV genotypes

When the data were stratified by HCV genotype, 55%(81/146) were infected with HCV genotype 1 (G1), and 45%(65/146) were infected with HCV non-G1 (15% with genotype 2, 8% with genotype 3, and 22% with genotype 6, respectively). The proportion of patients infected with HCV G1 who experienced NVR (51.9%) was significantly higher than in patients infected with HCV Non-G1 (30.8%). Compared with Non-G1 carriers, G1 carriers had a significantly higher risk of NVR (OR=2.42, 95% CI =1.22-4.80, P=0.01) (Table 3). Among patients infected with HCV G1, the association between NVR and the unfavorable rs8099917 with the TG+GG genotype was significant (P=0.009); however, in patients infected with HCV non-G1, the association was not observed (P=0.896).

**Table 3:** Treatment response according to IL28B (rs8099917) genotype in HCV G1 and non-G1 group.

Genotype	SVR(n=84)	NVR(n=62)	P value	OR（95%CI）
Non-G1	45(69.2%)	20(30.8%)		1.0(Reference)
TT	30	13	0.896	
TG+GG	15	7		
G1	39(48.1%)	42(51.9%)		2.42(1.22-4.80)
TT	25	13	0.003	
TG+GG	14	29		

G1, HCV Genotype 1; non-G1, Non HCV genotype 1.

### Univariate analyses of the factors correlated with HBV virological response and reactivation of HBV

Of the 146 dually-infected patients who completed the treatment and post-treatment follow-up evaluation. 67(45.9%) had detectable serum HBV DNA pretreatment, 79 Patients (54.1%) had undetectable levels. The rate of HBV viological response was signiﬁcantly different between patients with TT and TG+GG genotypes (57.9% vs. 31.0%, p=0.029). Moreover, the reactivation rate of HBV DNA with G variant (TG+GG) was signiﬁcantly higher than that of patients without the G variant (41.7% vs .13.9%, p=0.005). In addition, HBeAg-negative patients had a higher frequency of HBV response and a lower frequency of reactivation of HBV than HBeAg-positive patients (P=0.039 and P=0.017, respectively). However, There were no significant differences in the rate of HBV response or reactivation of HBV between HBV genotype B and C patients (P=0.187 and P=0.390 respectively) (Table 4).

**Table 4:** Univariate analyses of the factors correlated with HBV virological response and reactivation of HBV.

	HBV DNA detection status (n,%)		*P*-value
Patients with detectable serum HBV-DNA levels pretreatment (n=67)			
Posttreatment follow-up evaluation	HBV-DNA(+)	HBV-DNA(-)^※^	
rs8099917 genotype			
TT	16(42.1%)	22(57.9%)	0.029
TG+GG	20(69.0%)	9(31.0%)	
HBeAg state			
HBeAg Positive	17(89.5%)	2(10.5%)	0.039
HBeAg negative	30(62.5%)	18(37.5%)	
HBV genotype			
B	19(59.4%)	13(40.6%)	0.187
C	27(77.1%)	8(22.9%)	
Patients with undetectable serum HBV-DNA levels pretreatment (n=79)			
Posttreatment follow-up evaluation	HBV-DNA(+)^#^	HBV-DNA(-)	
rs8099917 genotype			
TT	6(13.9%)	37(86.1%)	0.005
TG+GG	15(41.7%)	21(58.3%)	
HBeAg state			
HBeAg Positive	10(55.6%)	8(44.4%)	0.017
HBeAg negative	14(22.9%)	47(77.1%)	
HBV genotype			
B	12(44.4%)	15(55.6%)	0.390
C	21(55.3%)	17(44.7%)	

+ HBV DNA detectable by a real-time PCR ( ≥200 IU/mL).

HBV DNA not detectable by a real-time PCR (≤200 IU/mL).

※ HBV virologic response was deﬁned as a reduction of serum HBV-DNA level to 200 IU/mL at the end of treatment in those with detectable serum HBV-DNA levels pretreatment;

# HBV virologic reappearance was deﬁned as an increase of posttreatment serum HBV-DNA levels to 200 IU/mL in those with pretreatment undetectable serum HBV-DNA levels.

### Multivariate logistic regression analyses of the factors correlated with HCV- NVR

To further examine the relative contribution of factors associated with NVR in HCV G1 and HCV non-G1 patients, we used a logistic regression model. When adjusting for baseline levels of HBV DNA and HCV RNA viral loads, HBeAg status, ALT, age, gender, BMI, we found that the G allele of rs8099917 was the most significant factor for predicting HCV-NVR in HCV G1 patients (OR = 10.2, 95% CI =2.51–41.7, P = 0.001), followed by HBV DNA viral load (OR = 3.22, 95% CI =2.46–5.50, P = 0.030). Full analysis suggested that the presence of rs8099917 G variant was a most important predictive factor of NVR before PEG-IFN-α/RBV therapy in HCV/HBV patients with the HCV-G1. Whereas, when adjusting for the same parameters, the rs8099917 G variant was not a signiﬁcant predictor of NVR in non-G1 patients (P=0.361) (Table 5).

**Table 5:** Factors associated with NVR by multivariate logistic regression.

	HCV genotype 1(NVR vs. SVR)			HCV non-genotype 1(NVR vs. SVR)		
	Odds ratio	95% CI	*P* value	Odds ratio	95% CI	*P* value
rs8099917 G variant	10.2	2.51-41.7	0.001	2.08	0.43-10.1	0.361
Age	1.02	0.96-1.06	0.623	1.01	0.94-1.07	0.852
Gender	1.69	0.51-5.60	0.388	0.62	0.13-3.04	0.559
BMI	1.24	0.98-1.56	0.064	0.97	0.74-1.29	0.856
HBV DNA	3.22	2.46-5.5	0.030	3.15	0.63-15.7	0.162
HBeAg State	1.45	0.41-5.14	0.563	6.17	1.03-36.9	0.046
HCV RNA	1.16	0.63-2.15	0.619	0.87	0.47-1.61	0.671

## Discussion

This report presents the results of a retrospective analysis of an HBV/HCV dually-infected cohort, who more frequently develop advanced liver disease, cirrhosis and HCC when compared to mono-infected patients. It is important to design and perform therapeutic trials to determine the best treatment for dually-infected patients. To our best knowledge, this is the first Chinese cohort study to examine the virological effects using IL28B polymorphisms in dual infection patients. Results of the current study indicate that patients with the rs8099917 TG+GG and rs12979860 TC+CC genotypes had a higher likelihood of NVR and a lower chance of SVR, similar to previous reports[17,26,27].This strategy could conceivably play a very important role in clinical setting. Adverse drug reactions could then avoided in patients unlikely to benefit from treatment, and the substantial cost of PEG-IFN-α/RBV treatment could be reduced.

Recently, many studies have confirmed the importance of different IL28B SNPs in the natural course and IFN-based treatment outcomes of HCV and HBV infection [16,17,28]. In the current study, we found that rs8099917 G variants (TG+GG) and rs12979860 T variants (CT+TT) carriers had a significantly higher risk of NVR ,similar to previously reported findings for HCV-mono-infected patients [16,26]. It is widely accepted that both host and virus factors can influence the treatment response [27,29,30]. However, there have been limited data regarding the prevalence of HCV genotypes and IL-28B SNP to their impact on outcomes in patients with HBV/HCV dual infections. Interestingly, when we analyzed the relationship between IL28B genotype and NVR separately for G1 and non-G1 subjects, we found that rs8099917 G variants (TG+GG) interact with HCV G1 to result in a higher risk of NVR. In contrast, the rate of NVR in patients infected with non-G1 types was not affected by rs8099917 genotype, suggesting that the predictive value of genotyping this SNP may limited to patients infected with G1. Nevertheless, the small number of non-G1 infected patients in our study prevented us from drawing conclusions about the predictive value. Overall, we showed that a combination of HCV genotype and IL-28B genotype may provide clinically powerful predictive values. IL28B SNP now may guide clinical decision making regarding whether or not to initiate therapy, how long to continue therapy, in order to avoid unpleasant side effects associated with treatment of HCV/HBV dually- infected patients.

Little is known about the HBV reactivation with PEG-IFN-α/RBV among HBV/HCV dually-infected patients. In this study, we found that the IL28 SNP genotype and HBeAg status were associated with HBV DNA reactivation. The reactivation rate of HBV DNA of the patients with G carriage (TG+GG) was significantly higher than those TT. Moreover, HBeAg-positive patients had a lower frequency of HBV response and a higher frequency of reactivation of HBV. These results were very similar to those reported in a large community-based follow-up study in HBV mono-infected patients [31,32]. Given the risk of HBV reactivation in G carriage or HBeAg-positive patients, clinicians should exercise caution when treating these patients. Therefore, close virological monitoring of HBV/HCV dual-infected patients after PEG-IFN-α/RBV therapies is absolutely necessary. 

Moreover, multivariate logistic regression analysis showed that G variant was an important predictive factor of HCV NVR in patients infected with HCV G1, but not in patients infected with HCV non-G1. In HCV G1 subgroup, HBV DNA level also contribute to HCV NVR, this results indicated that patients with G variant interact with high levels of HBV DNA might be able to achieve a high rate of HCV NVR. Intriguingly, we found that HBeAg status was an independent predictive factors of HCV NVR in HCV non-G1 subgroup, this findings indicated that HBeAg status might be more important to predict HCV NVR than genetic variation. However, it is possible that such a finding is attributable to the relatively small numbers of subjects in our cohort. Further studies of cohorts with large cases or different ethnic profiles will be necessary to determine whether there are differences between HCV G1 and non-G1 in dually-infected patients in regard to the predictive value of IL28B genotyping.

## Conclusion

All in all, our findings indicate that the IL28B polymorphism is a reliable predictor of PEG-IFN-α/RBV therapy for Chinese HCV/HBV dually-infected patients. Genotyping might be used for pretreatment stratification aimed at optimizing the treatment of this population. We recommend close virological monitoring of HCV/HBV dually-infected patients with unfavorable Il-28B genotypes.

## References

[1] Jamma S, Hussain G, Lau DT (2010) Current Concepts of HBV/HCV Coinfection: Coexistence, but Not Necessarily in Harmony. Curr Hepatol Rep 9: 260-269. doi:10.1007/s11901-010-0060-4. PMC3023453 21258658

[2] Pontisso P, Ruvoletto MG, Fattovich G, Chemello L, Gallorini A et al. (1993) Clinical and virological profiles in patients with multiple hepatitis virus infections. Gastroenterology 105: 1529-1533. doi:10.1016/0016-5085(93)90161-5. PubMed: 8224658. 8224658

[3] Crespo J, Lozano JL, de la Cruz F, Rodrigo L, Rodríguez M et al. (1994) Prevalence and significance of hepatitis C viremia in chronic active hepatitis B. Am J Gastroenterol 89: 1147-1151. PubMed: 8053425. 8053425

[4] Chu CJ, Lee SD (2008) Hepatitis B virus/hepatitis C virus coinfection: epidemiology, clinical features, viral interactions and treatment. J Gastroenterol Hepatol 23: 512-520. doi:10.1111/j.1440-1746.2008.05384.x. PubMed: 18397482. 18397482

[5] Li D, Long Y, Wang T, Xiao D, Zhang J et al. (2013) Epidemiology of hepatitis C virus infection in highly endemic HBV areas in China. PLOS ONE 8: e54815. doi:10.1371/journal.pone.0054815. PubMed: 23372775. 23372775 PMC3555996

[6] Bini EJ, Perumalswami PV (2010) Hepatitis B virus infection among American patients with chronic hepatitis C virus infection: prevalence, racial/ethnic differences, and viral interactions. Hepatology 51: 759-766. PubMed: 20140950. 20140950 10.1002/hep.23461

[7] Cacciola I, Pollicino T, Squadrito G, Cerenzia G, Orlando ME et al. (1999) Occult hepatitis B virus infection in patients with chronic hepatitis C liver disease. N Engl J Med 341: 22-26. doi:10.1056/NEJM199907013410104. PubMed: 10387938. 10387938

[8] Chiaramonte M, Stroffolini T, Vian A, Stazi MA, Floreani A et al. (1999) Rate of incidence of hepatocellular carcinoma in patients with compensated viral cirrhosis. Cancer 85: 2132-2137. doi:10.1002/(SICI)1097-0142(19990515)85:10. PubMed: 10326690. 10326690

[9] Liaw YF, Chen YC, Sheen IS, Chien RN, Yeh CT et al. (2004) Impact of acute hepatitis C virus superinfection in patients with chronic hepatitis B virus infection. Gastroenterology 126: 1024-1029. doi:10.1053/j.gastro.2004.01.011. PubMed: 15057742. 15057742

[10] Yu ML, Lee CM, Chen CL, Chuang WL, Lu SN et al. (2013) Sustained hcv clearance and increased HBsAg seroclearance in patients with dual chronic hepatitis C and B during post-treatment follow-up. Hepatology. 10.1002/hep.26266 23322699

[11] Liu CJ, Chuang WL, Lee CM, Yu ML, Lu SN et al. (2009) Peginterferon alfa-2a plus ribavirin for the treatment of dual chronic infection with hepatitis B and C viruses. Gastroenterology 136: 496-504 e493 doi:10.1053/j.gastro.2008.10.049. PubMed: 19084016. 19084016

[12] Liu CJ, Chen PJ, Lai MY, Kao JH, Jeng YM et al. (2003) Ribavirin and interferon is effective for hepatitis C virus clearance in hepatitis B and C dually infected patients. Hepatology 37: 568-576. doi:10.1053/jhep.2003.50096. PubMed: 12601355. 12601355

[13] Hung CH, Lee CM, Lu SN, Wang JH, Tung HD et al. (2005) Combination therapy with interferon-alpha and ribavirin in patients with dual hepatitis B and hepatitis C virus infection. J Gastroenterol Hepatol 20: 727-732. doi:10.1111/j.1440-1746.2005.03791.x. PubMed: 15853986. 15853986

[14] Hung CH, Lee CM, Lu SN, Wang JH, Chen CH et al. (2010) Hepatic steatosis with hepatitis B virus/hepatitis C virus dual infection. Hepatology 52: 1521-1522. doi:10.1002/hep.23740. PubMed: 20672387. 20672387

[15] Ge D, Fellay J, Thompson AJ, Simon JS, Shianna KV et al. (2009) Genetic variation in IL28B predicts hepatitis C treatment-induced viral clearance. Nature 461: 399-401. doi:10.1038/nature08309. PubMed: 19684573. 19684573

[16] Suppiah V, Moldovan M, Ahlenstiel G, Berg T, Weltman M et al. (2009) IL28B is associated with response to chronic hepatitis C interferon-alpha and ribavirin therapy. Nat Genet 41: 1100-1104. doi:10.1038/ng.447. PubMed: 19749758. 19749758

[17] Tanaka Y, Nishida N, Sugiyama M, Kurosaki M, Matsuura K et al. (2009) Genome-wide association of IL28B with response to pegylated interferon-alpha and ribavirin therapy for chronic hepatitis C. Nat Genet 41: 1105-1109. doi:10.1038/ng.449. PubMed: 19749757. 19749757

[18] Lampertico P, Viganò M, Cheroni C, Facchetti F, Invernizzi F et al. (2013) IL28B polymorphisms predict interferon-related hepatitis B surface antigen seroclearance in genotype D hepatitis B e antigen-negative patients with chronic hepatitis B. Hepatology 57: 890-896. doi:10.1002/hep.25749. PubMed: 22473858. 22473858

[19] Sonneveld MJ, Wong VW, Woltman AM, Wong GL, Cakaloglu Y et al. (2012) Polymorphisms near IL28B and serologic response to peginterferon in HBeAg-positive patients with chronic hepatitis B. Gastroenterology 142: 513-520 e511 doi:10.1053/j.gastro.2011.11.025. PubMed: 22108195. 22108195

[20] Aparicio E, Parera M, Franco S, Pérez-Alvarez N, Tural C et al. (2010) IL28B SNP rs8099917 is strongly associated with pegylated interferon-alpha and ribavirin therapy treatment failure in HCV/HIV-1 coinfected patients. PLOS ONE 5: e13771. doi:10.1371/journal.pone.0013771. PubMed: 21048934. 21048934 PMC2966433

[21] Ank N, West H, Bartholdy C, Eriksson K, Thomsen AR et al. (2006) Lambda interferon (IFN-lambda), a type III IFN, is induced by viruses and IFNs and displays potent antiviral activity against select virus infections in vivo. J Virol 80: 4501-4509. doi:10.1128/JVI.80.9.4501-4509.2006. PubMed: 16611910. 16611910 PMC1472004

[22] Ank N, West H, Paludan SR (2006) IFN-lambda: novel antiviral cytokines. J Interferon Cytokine Res 26: 373-379. doi:10.1089/jir.2006.26.373. PubMed: 16734557. 16734557

[23] Strader DB, Wright T, Thomas DL, Seeff LB (2004) Diagnosis, management, and treatment of hepatitis C. Hepatology 39: 1147-1171. doi:10.1002/hep.20119. PubMed: 15057920. 15057920

[24] Lok AS, McMahon BJ (2007) Chronic hepatitis B. Hepatology 45: 507-539. doi:10.1002/hep.21513. PubMed: 17256718. 17256718

[25] Desmet VJ, Gerber M, Hoofnagle JH, Manns M, Scheuer PJ (1994) Classification of chronic hepatitis: diagnosis, grading and staging. Hepatology 19: 1513-1520. doi:10.1002/hep.1840190629. PubMed: 8188183. 8188183

[26] Thompson AJ, Muir AJ, Sulkowski MS, Ge D, Fellay J et al. (2010) Interleukin-28B polymorphism improves viral kinetics and is the strongest pretreatment predictor of sustained virologic response in genotype 1 hepatitis C virus. Gastroenterology 139: 120-129 e118 doi:10.1053/j.gastro.2010.04.013. PubMed: 20399780. 20399780

[27] Guo X, Zhao Z, Xie J, Cai Q, Zhang X et al. (2012) Prediction of response to pegylated-interferon-alpha and ribavirin therapy in Chinese patients infected with different hepatitis C virus genotype. Virol J 9: 123. doi:10.1186/1743-422X-9-123. PubMed: 22713131. 22713131 PMC3439299

[28] Thomas DL, Thio CL, Martin MP, Qi Y, Ge D et al. (2009) Genetic variation in IL28B and spontaneous clearance of hepatitis C virus. Nature 461: 798-801. doi:10.1038/nature08463. PubMed: 19759533. 19759533 PMC3172006

[29] Yu ML, Chuang WL (2009) Treatment of chronic hepatitis C in Asia: when East meets West. J Gastroenterol Hepatol 24: 336-345. doi:10.1111/j.1440-1746.2009.05789.x. PubMed: 19335784. 19335784

[30] Georgel P, Schuster C, Zeisel MB, Stoll-Keller F, Berg T et al. (2010) Virus-host interactions in hepatitis C virus infection: implications for molecular pathogenesis and antiviral strategies. Trends Mol Med 16: 277-286. doi:10.1016/j.molmed.2010.04.003. PubMed: 20537953. 20537953

[31] Niederau C, Heintges T, Lange S, Goldmann G, Niederau CM et al. (1996) Long-term follow-up of HBeAg-positive patients treated with interferon alfa for chronic hepatitis B. N Engl J Med 334: 1422-1427. doi:10.1056/NEJM199605303342202. PubMed: 8618580. 8618580

[32] ter Borg MJ, van Zonneveld M, Zeuzem S, Senturk H, Akarca US et al. (2006) Patterns of viral decline during PEG-interferon alpha-2b therapy in HBeAg-positive chronic hepatitis B: relation to treatment response. Hepatology 44: 721-727. doi:10.1002/hep.21302. PubMed: 16941701. 16941701

[33] Hung CH, Lu SN, Wang JH, Lee CM, Chen TM et al. (2003) Correlation between ultrasonographic and pathologic diagnoses of hepatitis B and C virus-related cirrhosis. J Gastroenterol 38: 153-157. doi:10.1007/s005350300025. PubMed: 12640529. 12640529

