# Correction: Genetic Variation in Interleukin 28B and Response to Antiviral Therapy in Patients with Dual Chronic Infection with Hepatitis B and C Viruses

**DOI:** 10.1371/annotation/d042d8d6-cda8-4bdc-8ab4-821e08be0f1f

**Published:** 2013-11-07

**Authors:** Xiaoyan Guo, Guilin Yang, Jin Yuan, Peng Ruan, Mingxia Zhang, Xincun Chen, Boping Zhou

The order of affiliations for all authors is incorrect. The correct order of affiliations is: 1 Department of Infectious Diseases, The Third Affiliated Hospital of Sun Yat-Sen University, Guangzhou, China, 2 Institute of Hepatology, Shenzhen Third people’s Hospital, Shenzhen, China, 3 Key Laboratory of Tropical Disease Control (Sun Yat-sen University), Ministry of Education, Guangzhou, China. 

